# Supplementary material for: Photonic water dynamically responsive to external stimuli
Source: Nat Commun. 2016 Aug 30;7:12559. doi: 10.1038/ncomms12559 (PMC5013559; doi:10.1038/ncomms12559)
Supplement: Supplementary Information — Supplementary Figures 1-9 [file ncomms12559-s1.pdf]

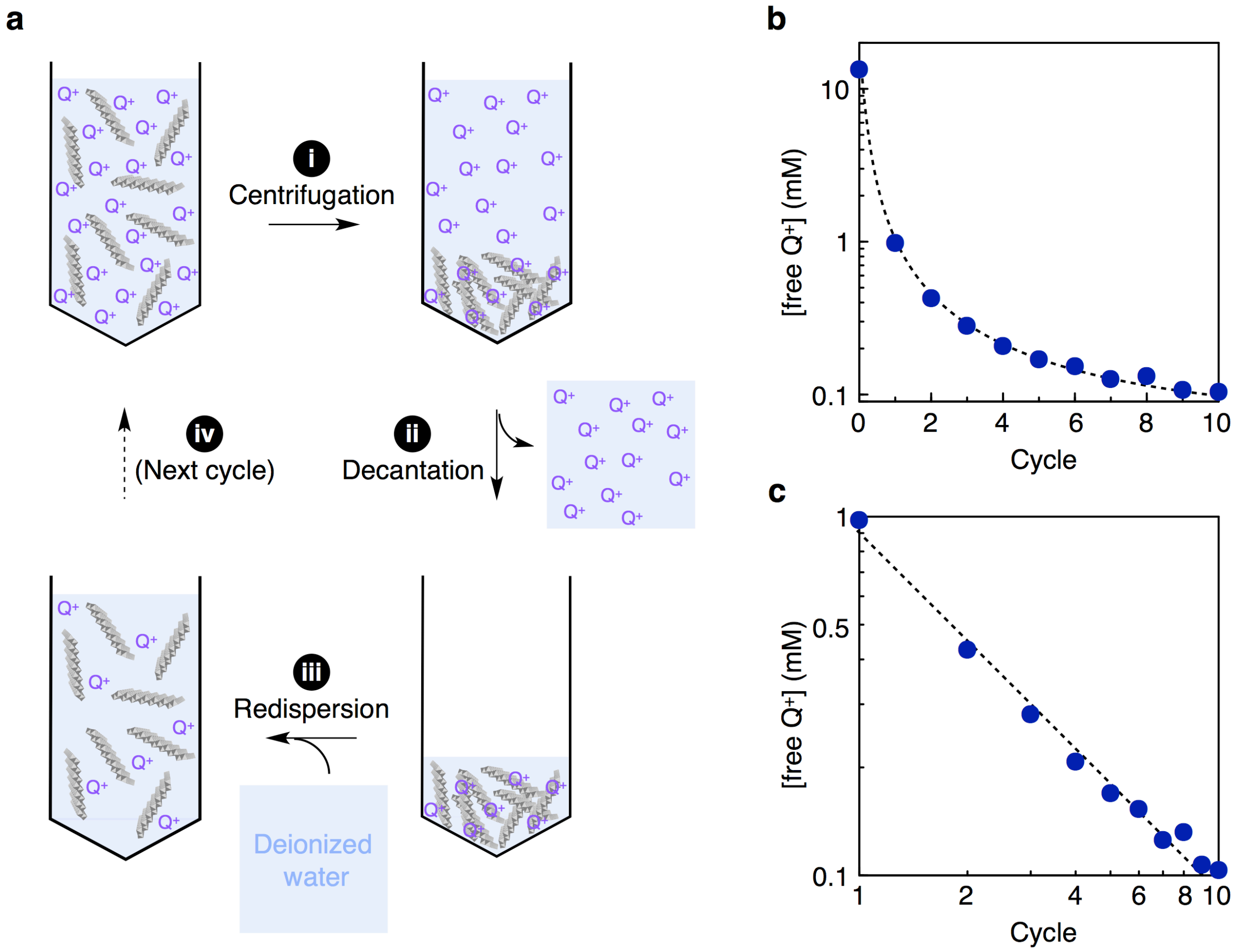

Supplementary Fig. 1 | Deionization of an aqueous dispersion of TiNSs. **a**, Schematic representation of the deionization process: (i) The original dispersion is centrifuged and separated into a supernatant and a sediment. (ii) The supernatant is removed by decantation. (iii) The sediment is re-dispersed in deionized water with the same amount as the removed supernatant. (iv) The resultant dispersion is used for the next deionization process up to ten cycles. **b**, Changes in the concentration of free Q^+ ions ([free Q^+]) in the aqueous dispersion of TiNSs upon repeating the deionization process, where [free Q^+] was estimated by the ion conductivity measurement. **c**, Double log plot of **b**.

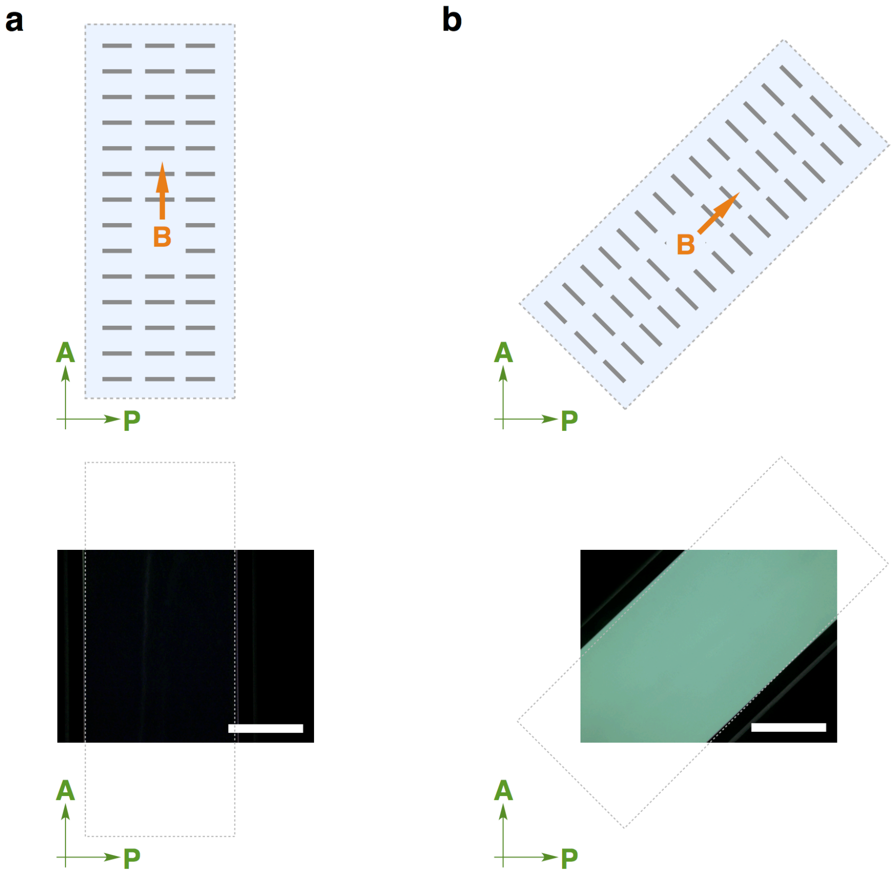

Supplementary Fig. 2 | Polarized optical microscopy (POM) observation of magnetically oriented photonic water. **a**, **b**, POM images of the magnetically oriented photonic water under crossed Nicols at 25 °C. For the procedure of sample preparation, see Methods in the main text (TiNS plane // viewing direction; sample thickness = 1.0 mm). Upon in-plane rotation of the cuvette, the image homogeneously changed its brightness and showed a contrast at every 45°. The image became darkest when the directions of the polarized light and applied magnetic field formed an angle of 0° or 90° (**a**) and turned brightest when the angle was 45° (**b**). Scale bars are 5 mm.

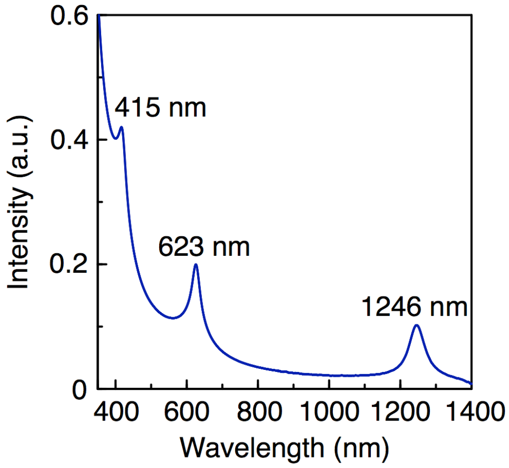

Supplementary Fig. 3 | Reflection spectrum of photonic water. A reflection spectrum of the magnetically oriented photonic water ([TiNS] = 0.13 vol%) at 25 °C. The primary (1246 nm), secondary (623 nm) and tertiary (415 nm) reflection peaks were observed, where the latter two peaks are the origin of purple color of the photonic water (Fig. 2c, iii). For the sample preparation, see Methods in the main text (TiNS plane ⊥ viewing direction; sample thickness = 1.0 mm).

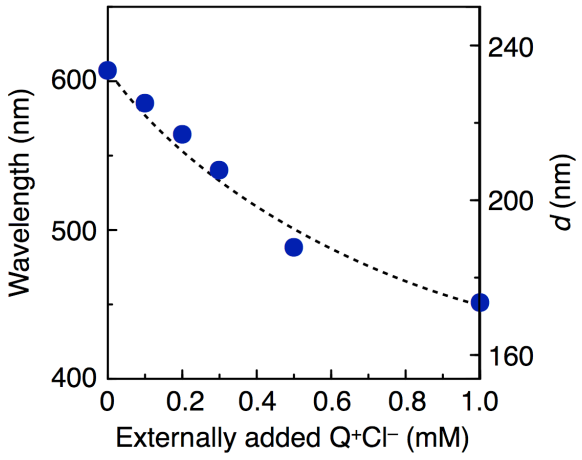

Supplementary Fig. 4 | Color modulation of photonic water in response to external ions. Changes in wavelength of the reflection peak in the reflection spectrum (left) and in the plane-to-plane distance between cofacial TiNSs (right) at 25 °C of the magnetically oriented photonic water ([TiNS] = 0.30 vol%) upon addition of Q^+Cl^-. For the sample preparation, see Methods in the main text (TiNS plane ⊥ viewing direction; sample thickness = 1.0 mm).

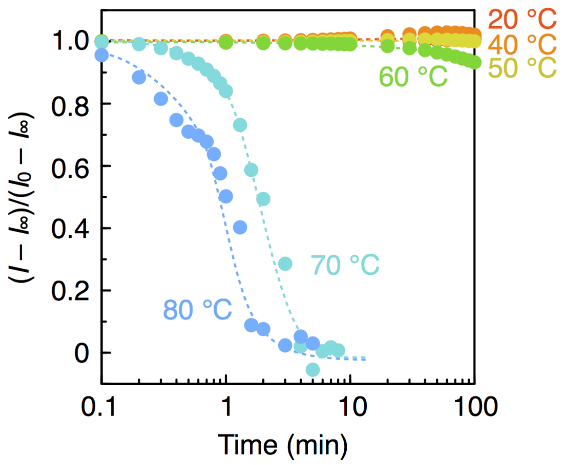

Supplementary Fig. 5 | Geometrical relaxation of photonic water at various temperatures. Relaxation profiles at 20–80 °C of magnetically ordered structures of the photonic water ([TiNS] = 0.30 vol%). For the sample preparation, see Methods in the main text (TiNS plane ⊥ viewing direction; sample thickness = 1.0 mm).

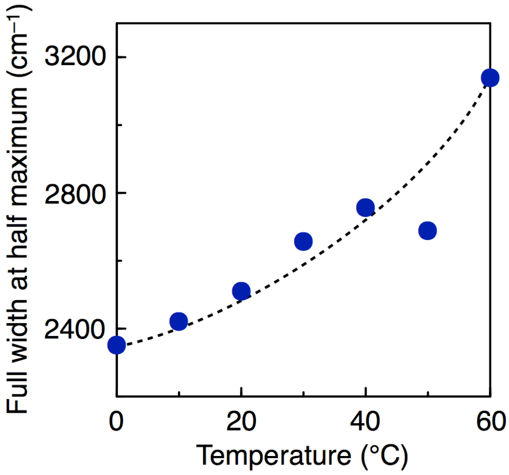

Supplementary Fig. 6 | Reflection peak width of photonic water at various temperatures. Full width at half maximum of the reflection peak of the magnetically oriented photonic water ([TiNS] = 0.30 vol%) at 0–60 °C (data from Fig. 5a, mid). For the sample preparation, see Methods in the main text (TiNS plane ⊥ viewing direction; sample thickness = 1.0 mm).

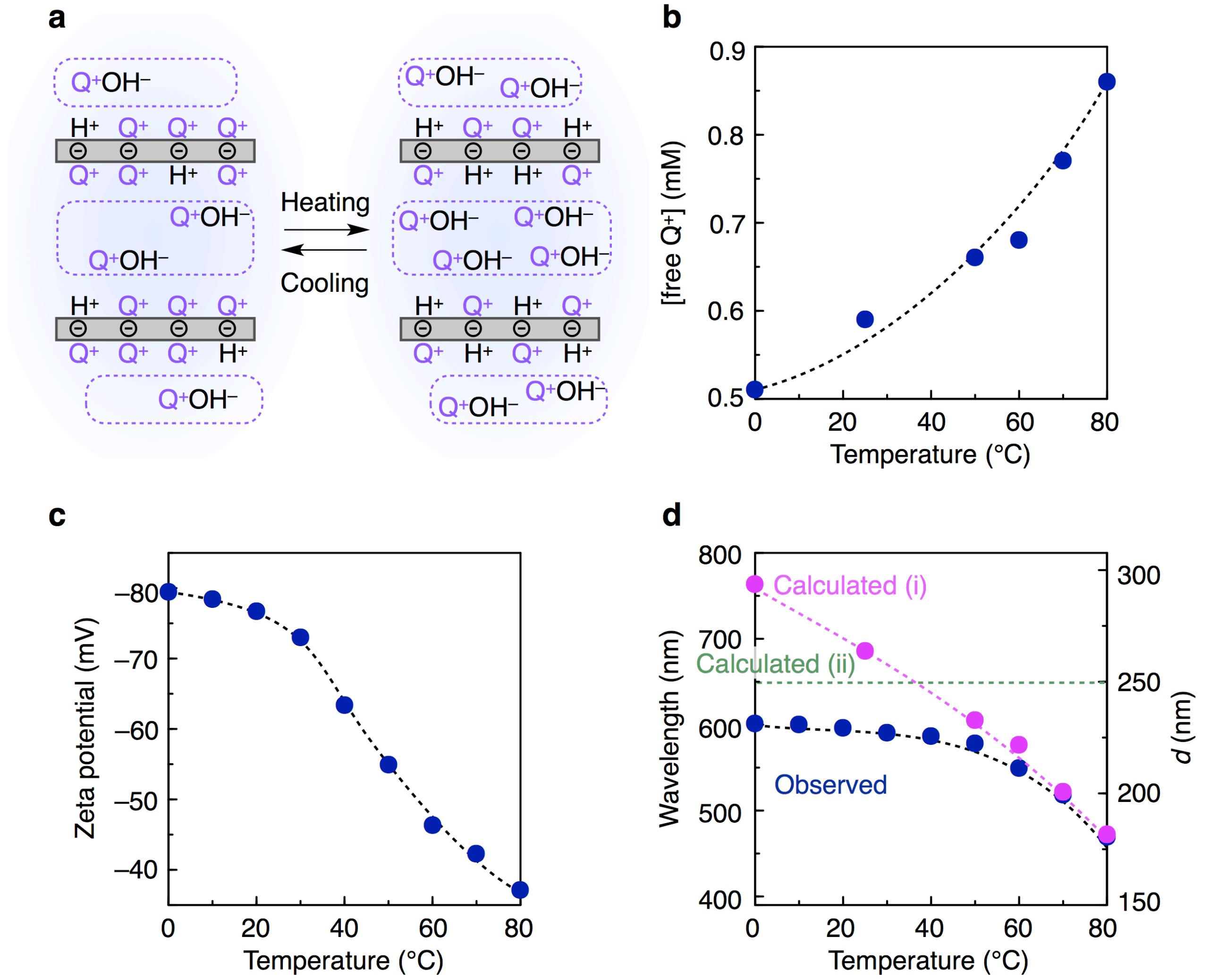

Supplementary Fig. 7 | Calculation of the periodic distances of TiNSs at various temperatures by using the DLVO theory. **a**, Schematic illustration of the thermoresponsive ionic-density change in TiNSs. **b**, Concentrations of free Q^+ ions at 0–80 °C. **c**, Zeta potentials of TiNS at 0–80 °C. **d**, Plots of the reflection wavelength and periodic distance (*d*) of TiNSs versus temperature: experimentally observed (data from Fig. 5a, navy), calculated from the DLVO theory (i, magenta) and calculated for a monodomain, homogeneous structure (ii, green).

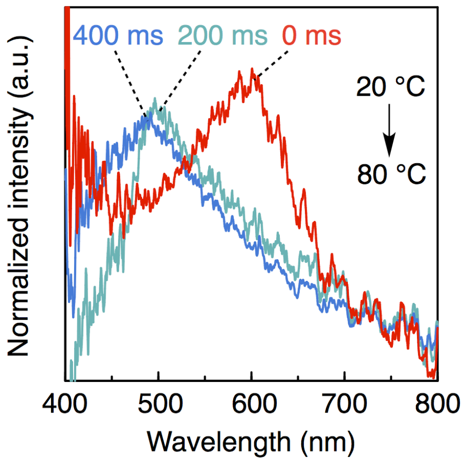

Supplementary Fig. 8 | Estimation for the color modulation rate of photonic water in response to a temperature change. Normalized reflection spectra of a photonic water droplet ([TiNS] = 0.30 vol%) that was abruptly heated from 20 °C to 80 °C. The spectra were taken at every 200 ms. For experimental details, see Methods in the main text.

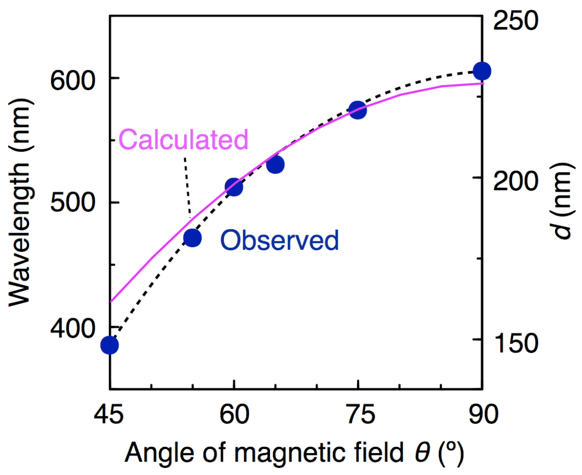

Supplementary Fig. 9 | Calculation of the color modulation of photonic water in response to the magnetic-field direction. Change in the reflection wavelength of the magnetically oriented photonic water ([TiNS] = 0.30 vol%) as a function of the angle of an externally applied magnetic field *θ*. Observed (data from Fig. 5b, navy) and calculated (magenta) data. For the sample preparation, see Methods in the main text (TiNS plane ⊥ viewing direction; sample thickness = 1.0 mm). For the calculation, a simple model of Bragg reflection was used.

$\lambda = 2\, n_{av}\, d \sin\theta$

λ: reflection wavelength

θ: grazing angle of the magnetic field with respect to the cuvette plane

d: plane-to-plane distance of cofacially oriented TiNSs (= 230 nm)

n_{water}: reflective index of water (= 1.3)

n_{TiNS}: reflective index of TiNS (= ~2.0)

ϕ_{water}: volume fraction of water (= 0.997)

ϕ_{TiNS}: volume fraction of TiNS (= 0.003)

n_{av}: averaged reflective index of the photonic water [= $(\phi_{water}\, n_{water}^2 + \phi_{TiNS}\, n_{TiNS}^2)^{0.5}$ = 1.3]
